# Detection of co-harboring OXA-58 and NDM-1 carbapenemase producing genes resided on a same plasmid from an *Acinetobacter pittii* clinical isolate in China

**DOI:** 10.22038/ijbms.2018.28934.6994

**Published:** 2019-01

**Authors:** Yili Chen, Penghao Guo, Han Huang, Yongxin Huang, Zhongwen Wu, Kang Liao

**Affiliations:** 1 Department of Laboratory Medicine, The First Afﬁliated Hospital of Sun Yat-sen University, 510080, Guangzhou, Guangdong, China; 2 Department of Laboratory Medicine, Zhongshan Medical School of Sun Yat-sen University, 510080, Guangzhou, Guangdong, China

**Keywords:** Acinetobacter pittii, Carbapenemase, Co-harboring, NDM-1, OXA-58

## Abstract

**Objective(s):**

*Acinetobacter pittii* has become an emerging opportunistic noscomial pathogen worldwide with multi-drug resistance. In the present study, an *A. pittii* strain was isolated from bronchoalveolar lavage fluid sample harboring both OXA-58 and NDM-1carbapenemase producing genes. The mechanisms of carbapenem resistance of the *A. pittii* strain was investigated.

**Materials and Methods:**

Carbapenemase producing genes were examined by PCR and DNA sequencing. S1-PFGE was used to localize carbapenemase encoding genes. Filter mating and electrotransformation were used to investigate the transferability of such carbapenemase encoding genes between different strains. Genetic surroundings of *bla*_OXA-58_ and *bla*_NDM-1_ genes were detected as well.

**Results:**

The *A. pittii* strain, carrying both OXA-58 and NDM-1 carbapenemase encoding genes, was resistant to all β-lactam antibiotics, while suscepitible to ciprofloxacin, levofloxacin, tobramycin, cotrimoxazole and tigecycline. Southern blot hybridization for the *bla*_OXA-58_ and *bla*_NDM-1_ gene indicated that the two genes locate in the same plasmid with molecular weight of 310.1-336.5kb. *Bla*_OXA-58_ was located in an IS*Aba3*-*bla*_OXA-58_-IS*Aba*3-like structure, and the blaNDM-1 gene cluster was embedded in an IS*Aba125*-*aphA6*- *bla*_NDM-1_*-ble*_MBL_*-ΔtrpF-dsbC-cutA* structure sequentially.

**Conclusion:**

In the present study, it is first reported an *A. pittii* clincal strain in China, co-harboring OXA-58 and NDM-1 carbapenemase producing genes residing on a same plasmid. In hospital and community settings, it is of great significance and urgence to increase the surveillance of these kinds of organisms.

## Introduction

*Acinetobacter spp. *are frequent pathogens responsible for nosocomial infections, including *Acinetobacter baumannii *as the predominant species followed by *Acinetobacter pittii* and *Acinetobacter nosocomialis *(1)*. *Since the phenomenon of carbapenem resistance increasingly emergences, there is a big challenge of multidrug resistant *Acinetobacter* in treating hospital infections (2, 3).

To our knowledge, the increasing expression of OXA type carbapenemase genes is the most frequent mechanism of carbapenem resistance in *Acinetobacter*. It mainly includes the intrinsic *bla*_OXA-51_*-like* gene as well as the horizontally acquired genes such as *bla*_OXA-23_*-like*, *bla*_OXA-24_*-like* and *bla*_OXA-58_*-like* genes (4, 5). The *bla*_OXA-23_*-like* genes are much more prevalent than the *bla*_OXA-24_*-like* and *bla*_OXA-58_*-like* genes. All of them are capable of yielding carbapenem resistance in a high level and result in serious local outbreaks (6-8). Moreover, the expression of metallo-β-lactam (MBL) carbapenemase also plays an important role in carbapenem resistance of *Acinetobacter*, including IMP, VIM, SIM, and NDM detected previously in *Acinetobacte*r (9). Although these MBLs genes were less detected than OXAs, their carbapenemase activities were typically much higher (10, 11). Especially, since 2008, there have been an increasing number of reports about the dissemination of NDM-1-producing *Acinetobacter spp *in many countries and it resulted in a major threat for clinical treatments in view of its highly frequent co-occurrence with other resistance genes (12-14). Recently, the global spread of bla*NDM-1*-harboring *A. pittii *strains is fierce. With these powerful genes, as the reservoirs for dissemination, they are able to transfer highly across various bacterial species (15-17). 

In this study, we investigated the antibiotic susceptibility, genetic environment, and transferability of a single clinical *A. pittii *isolate co-harboring *blaNDM-1 *and *blaOXA-58 *genes on a same plasmid, in order to improve awareness of the urgency of carbapenemase-producing *A. pittii* isolates in China.

## Materials and Methods

***Bacterial isolates***

The *A. pittii* was isolated from a bronchoalveolar lavage fluid sample of a 56-year-old man suffering from chronic obstructive pulmonary disease (COPD). It was initially identified as *Acinetobacter calcoaceticus baumannii *by Vitek 2 system (bioMérieux, Marcy l’Etoile, France). To confirm the identity of this strain, a fragment of the 16S rRNA gene was amplified using primers 27-forward (5’- AGA GTT TGA TCC TGG CTC AG -3’) and 1492-reverse (5’- GGT TAC CTT GTT ACG ACT T -3’) by PCR[10] and the resultant PCR product sequenced as *A. pittii* with 100% identity*, *designated as AB34.

***Antimicrobial susceptibility testing***

The minimum inhibitory concentration (MIC) of various antibiotics was detected on the Vitek 2 system (bioMérieux, Marcy l’Etoile, France). 

According to the CLSI clinical breakpoints (2017; CLSI Document M100-S27), antimicrobial susceptibility was interpreted. The following antibiotics were investigated in the present study: amikacin, ciprofloxacin, ampicillin/sulbactam, ceftazidime, imipenem, cotrimoxazol, tobramycin, piperacillin/tazobactam, cefepime, gentamicin, levofloxacin, meropenem, rifampin and tigecycline. Quality control for the MIC analysis was carried out with *Pseudomonas aeruginosa *ATCC 27853 and *Escherichia coli *ATCC 25922. 

***Screening of carbapenemases encoding genes***

Amplification of carbapenemases encoding genes was perfomed, including OXA-51, OXA-23, OXA-58, OXA-143, OXA-24, NDM-1, GIM-1, SPM-1, IMP-1, SIM-1, VIM-1. These primers are shown in Table 1. All amplicons were sequenced using the ABI PRISM Big Dye Terminator Cycle Sequencing Ready Reaction kit (Applied Biosystems -2- Inc, USA).

***S1-PFGE and southern blot***

The total bacterial DNA was first prepared in agarose plugs, digested with S1 nuclease (Takara, Japan) and further separated by PFGE, as reported previously (21). The DNA fragments were transferred horizontally to a nylon membrane (Millipore, USA), hybridized with digoxigen in-labeled bla_OXA-58_ and bla_NDM-1_ probe and detected using a nitroblue tetrazolium/5-bromo-4-chloro-3’-indolyl-phosphate color detection kit (Roche Applied Sciences, USA).

***Filter mating experiment***

Filter mating experiment was performed with the rifampin-resistant *EC600 *and azide-resistant *E. coli J53 *as the recipient strains. The transconjugants were selected on Mueller-Hinton agar plates containing [ampicillin (50 mg/l) and rifampicin (1024 mg/l)] or [ampicillin (50 mg/l) and NaN_3_ (200 mg/l)], respectively, and incubated for 16–18 hr at 37 ^°^C. The successful transconjugants were selected on Mueller-Hinton agar incorporating the same concentration of antibiotics mentioned above. The transformants would be confirmed the resistant genes by PCR.

***Plasmid construction and electrotransformation***

The plasmid DNA of isolate AB34 was extracted, digested by restriction enzyme *EcoRI* or *SacI* and then cloned into the cloning vector pPet28a**. **The conjugant was electrotransformed to *E. coli DH5α *competent cells and selected on Mueller-Hinton agar plates containing ampicillin (50 mg/L) in order to obtain the *E. coli* clone expressing the corresponding carbapenemase enzyme.

***Genetic surroundings detection***

A total genomic sample of *A. pittii* strain AB34 was extracted and purified using the Wizard Genomic DNA purification kit (Promega Corporation, Madison, WI) according to the manufacturer’s instructions. DNA concentration was estimated using a Qubit dsDNA HS Assay Kit and a Qubit 3.0 Fluorometer (Invitrogen, Thermo Fisher Scientific, USA). Extracted DNA was then sequenced with a standard 2×125 paired-end runs protocol on an Illumina HiSeq 2000 (Illumina, San Diego, CA, USA). The quality of the high-throughput sequence data was assessed by FastQC (http://www.bioinformatics.babraham.ac.uk/projects/fastqc/). Raw sequence reads were then *de novo* assembled using

Plasmid SPAdes 3.9.0 (http://bioinf.spbau.ru/spades)(22), in order to identify plasmid contigs, and the quality was assessed by QUAST (http://quast.bioinf. spbau.ru). 

***Nucleotide sequence accession numbers***

The *bla*_OXA-58_ or *bla*_NDM-1_ nucleotide sequences are available in GenBank under the accession number KF208466 or KF208467, respectively.

## Results

***Susceptibility results***

The isolate AB34 was resistant to all β-lactams including ceftazidime, cefepime and carbapenems as well as ampicillin/sulbactam inhibitor combinations, but remained susceptible to cotrimoxazole, tobramycin, ciprofloxacin, levofloxacin and tigecycline (Table 2).

***Detection of carbapenemases encoding genes***

Only OXA-58(599bp) and NDM-1(720bp) were detected in this strain. The amplified products were confirmed by sequencing. BLAST version 2.2.24 (http://blast.ddbj.nig.ac.jp/) was used to process the sequencing data and identify genes.

***Location of bla***_OXA-58_ ***and bla***_NDM-1_ ***genes***

**Figure 1:**
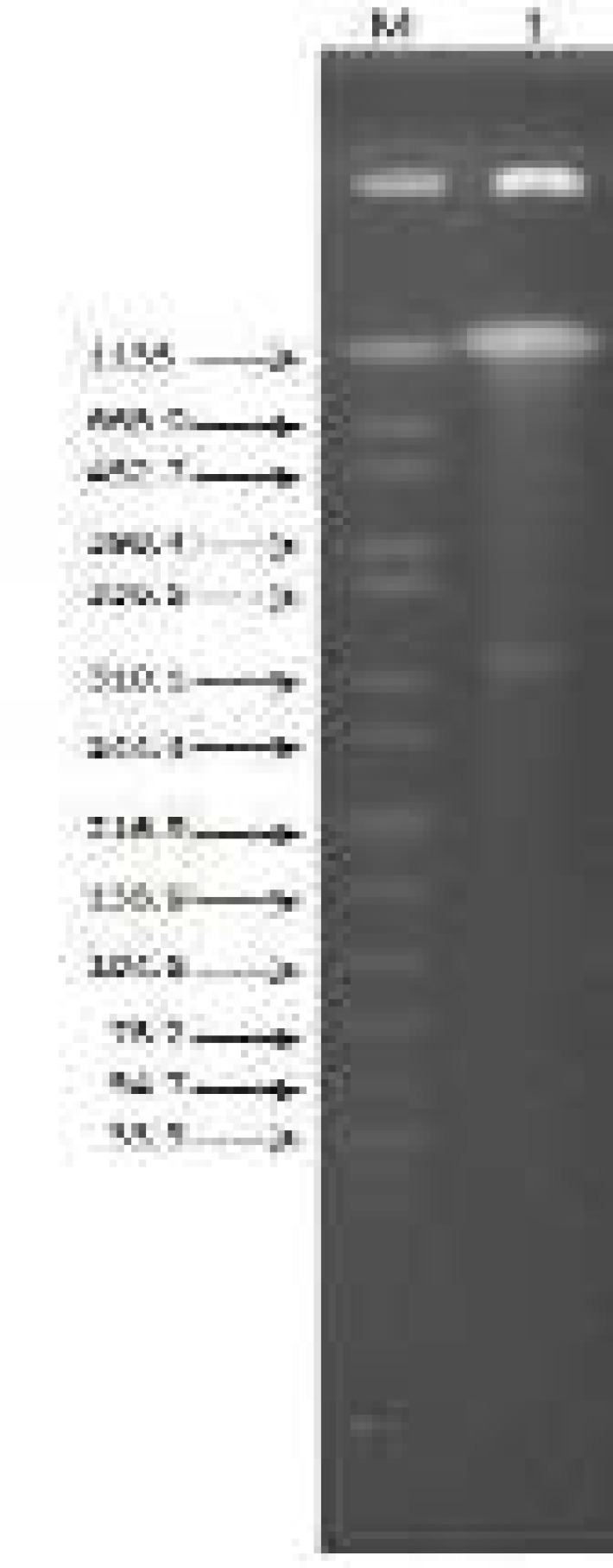
S1-PFGE. Lane M, DNA Marker; lane 1, strain AB34

**Figure 2:**
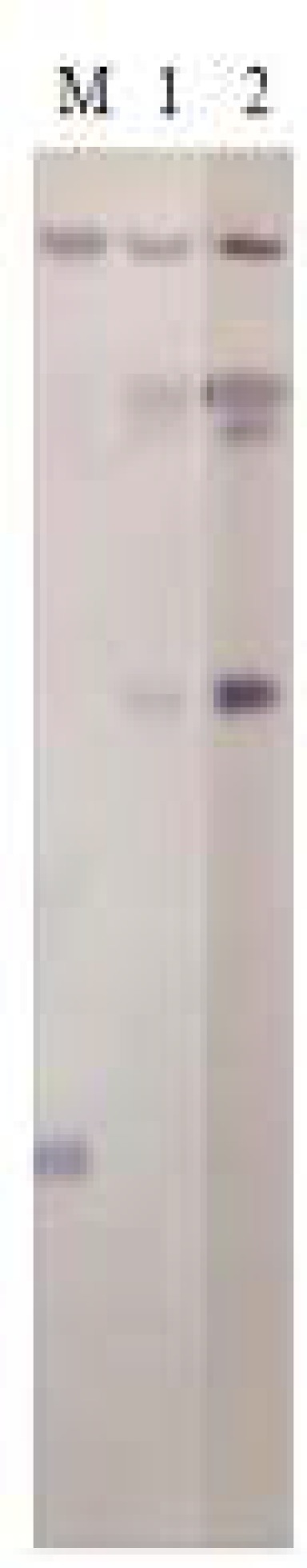
Southern blot hybridization of OXA-58 and NDM-1 genes. Lane 1, OXA-58 hybridization; lane 2, NDM-1hybridization; lane M, DNA Marker

After PFGE (Figure 1) and southern blot hybridization (Figure 2), it was found that both *bla*_OXA-58_ and* bla*_NDM-1_ genes resided on a same 310.1-336.5kb plasmid. Horizontal transfer of the two carbapenem resistance determinants from AB34 to *EC600 *or *E. coli J53* was not detected in filter mating.

***Genetic surroundings***

**Figure 3:**
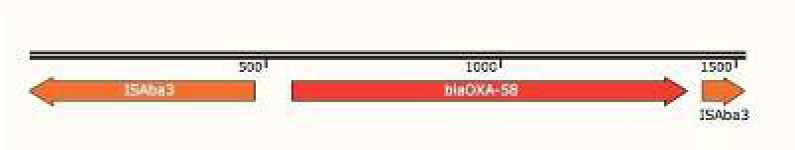
Analysis of bla_OXA-58_-carrying composite transposon in *Acinetobacter Pittii *AB34 genome. Genes and transcription orientations are indicated by arrows

**Figure 4:**
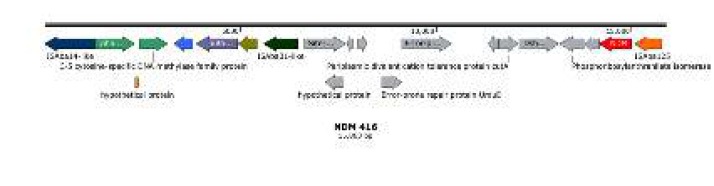
Analysis of bla_NDM-1_-carrying composite transposon in *Acinetobacter Pittii* AB34 genome. Genes and transcription orientations are indicated by arrows

A BLAST search against all completely sequenced *blaOXA-58* and* blaNDM-1*-genes-co-harboring plasmids in GenBank (http://www.ncbi.nlm.nih.gov/GenBank/) showed that the *blaOXA-58 *gene in AB34 was located down-stream of the shortened IS*Aba*3 gene, and up-stream of a complete copy of IS*Aba*3 gene (Figure 3). The *blaNDM-1* gene cluster of AB34 was arranged sequentially as IS*Aba*125, *aphA6*, *blaNDM-1*, *ble*MBL, ΔtrpF, *dsbD*,* cutA1*, *GroES*, *UmuD*, hypothetical protein, site-specific DNA methylase-like, IS*Aba*31-like, dihydrofolate reductase putative membrane protein, C-5 cytosine-specific DNA methylase family protein, IS*Aba*14-like from right to left (Figure 4).

## Discussion

Since the last decade, carbapenemase-producing *Acinetobacter spp* have disseminated rapidly throughout the world, posing an urgent threat to public health (1, 23). *A. pittii*, formerly named *Acinetobacter genomic* species 3, is increasingly recognized as a clinically important pathogen within the *Acinetobacter calcoaceticus– A. baumannii complex*, which addresses a particular concern due to its competency to acquire multidrug resistance against a wide range of antimicrobial agents (24).

**Table 1:** Primer sequences of the carbapenemases encoding genes

Target genes	Name of primers	Sequence	Amplicon size	**Reference**
OXA-51-live	OXA51_Mup	5'-TAATGCTTTGATCGGCCTTG-3'	353	(18)
	OXA51_Mdw	5'-TGGATTGCACTTCATCTTGG-3'		
OXA-23-live	OXA23_Mup	5'-GATCGGATTGGAGAACCAGA-3'	501	(18)
	OXA23_Mdw	5'-ATTTCTGACCGCATTTCCAT-3'		
OXA-24-live	OXA24_Mup	5'-GGTTAGTTGGCCCCCTTAAA-3'	246	(18)
	OXA24_Mdw	5'-AGTTGAGCGAAAAGGGGATT-3		
OXA-58-live	OXA58_Mup	5'-AAGTATTGGGGCTTGTGCTG-3'	599	(19)
	OXA58_Mdw	5'-CCCCTCTGCGCTCTACATAC-3'		
OXA-143-live	OXA143_Mup	5'-TGGCACTTTCAGCAGTTCCT-3'	149	(20)
	OXA143_Mdw	5'-TAATCTTGAGGGGGCCAACC-3'		
SIM	SIM-F	5'-TACAAGGGATTCGGCATCG-3'	570	(18)
	SIM-R	5'-TAATGGCCTGTTCCCATGTG-3'		
SPM	SPM-F	5'-AAAATCTGGGTACGCAAACG-3'	271	(18)
	SPM-R	5'-ACATTATCCGCTGGAACAGG-3'		
GIM	GIM-F	5'-TCGACACACCTTGGTCTGAA-3'	477	(18)
	GIM-R	5'-AACTTCCAACTTTGCCATGC-3'		
IMP	IMP-F	5'-GAAGGCGTTTATGTTCATAC-3'	587	(18)
	IMP-R	5'-GTACGTTTCAAGAGTGATGC-3'		
VIM	VIM-F	5'-GTTTGGTCGCATATCGCAAC-3'	389	(18)
	VIM-R	5'-AATGCGCAGCACCAGGATAG-3'		
NDM-1	NDM-F	5'-GCAGCTTGTCGGCCATGCGGGC-3'	782	(18)
	NDM-R	5'-GGTCGCGAAGCTGAGCACCGCAT-3'		

**Table 2:** MIC for the *Acinetobacter pittii *AB34

Antibiotics	MIC（μg/mL）	Interpretation
amikacin	24	I
ciprofloxacin	0.19	S
imipenem	≥32	R
cotrimoxazole	<=20	S
tobramycin	<=1	S
ampicillin/sulbactam	≥32	R
gentamicin	≥256	R
levofloxacin	2	S
ceftazidime	≥64	R
cefepime	≥256	R
meropenem	≥32	R
tigecycline	2	S

The first known OXA-58-producing *Acinetobacter *strain was isolated in France in 2003 (9). It shares less than 50% of amino-acid homology with oxacillinase. OXA-58 is a widely spread carbapenem-hydrolyzing class D β-lactamases (CHDLs) that has been reported in *Acinetobacter spp*. from Europe (25), Argentina (26), Australia (27), the United States (28) and many Asian countries (29). Though OXA-58 shows only low carbapenem-hydrolyzing activity *in vitro*, the insertion sequence upstream of *bla*_OXA-58_ enhances its transcription greatly and mediates resistance to carbapenems (30-32). It is speculated that the insertion of other IS element into IS*Aba3*-like could generate a hybrid promoter to enhance the transcription of *bla*_OXA-58_ and mediate greater carbapenem resistance than the intact IS*Aba3*-like element as previously reported (32, 33). However, in China, the most common carbapenamase-producing type of *A. baumannii* is OXA-23,while OXA - 58 is rarely reported (34).

The NDM-1 gene encodes an enzyme that hydrolyses and inactivates all β-lactam antibiotics including carbapenems, except for aztreonam, and thus induces resistance to carbapenems (35). *A. baumannii *carrying NDM-1 have been reported from clinical and environmental isolates in several countries (36-39). Not only *Acinetobacter spp.* act as reservoirs for *blaNDM* genes in non-human settings, as recently shown in several Chinese studies with identification of NDM-1-producers among *A. calcoaceticus* and *Acinetobacter junii *from environmental samples from livestock farms (40), *Acinetobacter johnsonii* from hospital sewage (40) and *Acinetobacter lwoffii* from chickens(40), but also act as a source of *bla*_NDM_ genes then horizontally transferred to enterobacterial species as evidenced (41).

It is noteworthy that coexistence of *blaNDM* and *blaOXA* has been described in *Acinetobacter,* e.g. *blaOXA-23* and *blaNDM-1* in *A. baumannii* from India (42) and the Czech Republic (43), and *bla*_NDM-1_, *bla*_OXA-23_ and *bla*_IMP_ in *A. baumannii *from China (44). However, it remains unclear whether and how these co-existing carbapenemase genes are expressed to contribute to drug resistance. 

*A. pittii *44551 was recovered from a patient with gout combined with tuberculosis and was found to harbor the carbapenemase genes *bla*_NDM-1_ and *bla*_OXA-58_ on two different plasmids pNDM-44551 and pOXA58-44551, respectively, from China in 2015 (1). Emergence of ST119 *A. pittii *AP 882 co-harbouring NDM-1 and OXA-58 in Malaysia was reported as well, of which genes encoding NDM-1 and OXA-58 resided on an ca.140 kb mega plasmid and a 35 kb plasmid, respectively (45). 

Similarly, in our present study, AB34 was isolated and detected co-harbouring OXA-58 and NDM-1carbapenemase producing resided on the same 310.1-336.5kb plasmid. However, horizontal transfer of carbapenem resistance determinants from AB34 to *EC600 *or* E. coli J53* (AzR) was not detected in filter mating experiment. The up-stream and down-stream of OXA-58 gene in AB34 are ISAba3, which shows 99% similarity to *A. pittii *pOXA-58-44551 (1). It is reported that the structure of OXA-58 of *A. pittii *44551 is 372F-*ISAba3*-like-*bla*_OXA-58_-*ISAba3*, where the *bla*_OXA-58_ contributed little to β-lactams resistance due to a lack of the *blaOXA-58*-driven promoter (1). An intact *ISAba3*-like element upstream of *bla*_OXA-58_ has been linked to a lower level of resistance to imipenem compared with *bla*_OXA-58_ with hybrid promoters such as IS6 family-IS*Aba3*-like-*bla*_OXA-58_.

The upstream of NDM-1 of AB34 is IS*Aba*125, while the down-streams are arranged sequentially as *aphA6*, *bla*_NDM-1_, *ble*MBL, ΔtrpF, *dsbD*,* cutA1*, *GroES*, *UmuD*, hypothetical protein, site-specific DNA methylase-like, IS*Aba*31-like, dihydrofolate reductase putative membrane protein, C-5 cytosine-specific DNA methylase family protein, IS*Aba*14-like, which was with 99% sequence identity against that of *Acinetobacter lwoffii *pNDM-BJ01 from Beijing, China (46). It is proved that the genetic surroundings of *bla*_NDM-1_ is an important vector to mediate to integration and transfer. It should be noted that the IS*Aba125* element upstream of *bla*_NDM-1_ is usually intact in *Acinetobacter* but often truncated in *Enterobacteriaceae*, suggesting the probable spread of the *bla*_NDM-1_ genetic platforms from *Acinetobacter* to *Enterobacteriaceae *(47-50).

## Conclusion

This study has improved awareness of the urgence of carbapenemase-producing *A. pittii* isolates in China. Further investigations on the comparative genomic analysis of a large-scale sampling of *A. pittii *strains from a wide spatial and temporal range in the context of genomic epidemiological characteristics are currently on the way. These data highlight the molecular mechanisms contributing to the rapid development of antimicrobial resistance and will facilitate to expand our understanding of the global public health concern caused by *Acinetobacter spp*.
